# Pre‐ and Post‐Evaluation of the Reach and Impact of a Nationwide Media Campaign to Promote Nutritional Services and Preventive Measures in Ethiopia: A Two‐Round Mobile Survey

**DOI:** 10.1002/hsr2.72835

**Published:** 2026-07-16

**Authors:** Feleke Doyore Agide, Beimnet Desalegn Kedida, Zeamanuel Anteneh Yigzaw, Yewubdar Hirpa, Eneyew Talie Fenta, Hiwot Getachew, Rachana Sharma, Nardos Birru, Stanley Chitekwe, Mussie Tefera, Finina Abebe Ware, Dessie Abebaw, Tolasa Yadate

**Affiliations:** ^1^ Department of Public Health, School of Allied Health Sciences Kampala International University Kampala Uganda; ^2^ Department of Health Education and Health Promotion, School of Public Health, College of Medicine and Health Sciences Wachemo University Hossana Ethiopia; ^3^ School of Public Health, College of Medicine and Health Sciences Wolaita Sodo University Sodo Ethiopia; ^4^ Department of Health Promotion and Behavioral Sciences, School of Public Health, College of Medicine and Health Sciences Bahir Dar University Bahir Dar Ethiopia; ^5^ Federal Ministry of Health Addis Ababa Ethiopia; ^6^ Department of public health, College of medicine and health science Injibara University Injibara Ethiopia; ^7^ SBC Specialist, UNICEF Addis Ababa Ethiopia; ^8^ SBC Manager, UNICEF Addis Ababa Ethiopia; ^9^ Nutrition Specialist, UNICEF Addis Ababa Ethiopia; ^10^ Chief Nutrition, UNICEF Addis Ababa Ethiopia; ^11^ Country Manager Viamo Ethiopia; ^12^ Clinton Health Access Initiative (CHAI) Addis Ababa Ethiopia; ^13^ Department of Epidemiology and Biostatistics, Institute of Public Health, College of Medicine and Health Sciences University of Gondar Ethiopia; ^14^ School of Public Health, College of health Sciences and Medicine Dilla University Dilla Ethiopia

**Keywords:** Ethiopia, interactive voice response, media, nutrition, random digit dialing

## Abstract

**Background and Aims:**

The practice of health promotion intervention program planning largely depends on program development and evaluation. Program developed through social media and electronic media have brought about significant changes in nutrition‐related problems in the world. Therefore, this study was aimed at assessing on promoting preventive measures of nutritional services in Ethiopia.

**Methods:**

This two‐round mobile survey used post‐comparison group evaluation design using Random Digit Dialing and an Interactive Voice Response. A total of 2704 participants completed survey at baseline and end‐line after the nutritional intervention. Then, a pre‐ and post‐evaluation data was collected using random digit dialing and an interactive voice response. To evaluate the media campaign's impact, the study examined outcomes related to nutritional knowledge, attitudes, and practices using post‐intervention comparison data. A propensity score matching analysis was used.

**Results:**

Among the 2704 mobile subscribers who completed the survey questions, 88% versus 86% reported as knowledgeable, and 57% versus 52% had positive towards nutritional practice at end‐line and baseline, respectively. The propensity score matching analysis showed that exposure to the media campaign was significantly associated with knowledge of deworming (proportion difference, 0.30; 95% CI, 0.20, 0.49; *p* < 0.001, attitude of growth monitoring (0.70; 95% CI, 0.42, 0.99; *p* < 0.001), attitude of vitamin A (0.50; 95% CI, 0.20, 0.91; *p* < 0.001) and overall practice of Iron and folic acid (0.04; 95% CI, 0.02, 0.09; *p* < 0.0001).

**Conclusion:**

The study highlights a strong synergistic relationship between media campaign interventions and the utilization of nutritional services. These findings also provide valuable insights for informing the design and implementation of future media campaigns in Ethiopia. To enhance the effectiveness of such interventions, field experts should address limitations in media reach and optimize exposure intensity through broader audience coverage and more frequent dissemination of campaign messages.

## 1 Introduction

Globally, malnutrition remains one of the most pressing public health challenges. According to the World Health Organization (WHO), an estimated 149 million children under five are stunted, 45 million are wasted, and 37 million are overweight [1]. Micronutrient deficiencies, often termed “hidden hunger,” affect billions of people worldwide, with nearly half of all deaths among children under five linked to undernutrition. These figures underscore the pressing need for scalable and cost‐effective interventions to enhance awareness of nutrition and preventive health services [2].

In Ethiopia, despite encouraging progress, malnutrition continues to pose a major barrier to child survival and human capital development. The 2022 Ethiopian Demographic and Health Survey (EDHS) reported that 37% of children under five experienced stunting, 7% experienced wasting, and 21% were underweight [3]. Moreover, coverage of essential preventive services remains suboptimal: vitamin A supplementation (43%), deworming (36%), and iron–folic acid supplementation during pregnancy (less than 20%) all fall short of national targets. These gaps lead to preventable morbidity, stunting and poor maternal health, highlighting the need for innovative large‐scale strategies in addressing persistent malnutrition [1, 3].

In addressing these challenges, the Ethiopian government and development partners have implemented various approaches, including the Health Extension Program, community‐based nutrition interventions, and mass media campaigns focused on improving knowledge and service uptake [4, 5]. Evidence from other low‐ and middle‐income countries demonstrates that media interventions can increase awareness, shift attitudes, and promote the utilization of health and nutrition services [6, 7]. Ethiopia has similarly prioritized mass media campaigns as cost‐effective strategies for reaching millions of people with timely, evidence‐based messages [8].

Nevertheless, despite these investments, limited evidence exists regarding the actual reach and effectiveness of nationwide media campaigns in Ethiopia. Most previous evaluations have focused on small‐scale or community‐based interventions, leaving a gap in understanding the broader impact of media‐driven approaches [9]. This is especially essential in Ethiopia, where malnutrition persists and is compounded by ongoing issues like as conflict, drought, and inflation [10].

Mass media is well regarded as a potent tool for health promotion and illness prevention, with reach, intensity, and influence all playing important roles in altering health behaviors [11, 12]. Communication techniques for preventative health include balanced‐diet messages, supplements promotion, and early childhood nutrition initiatives [13]. The WHO also underlines the significance of these treatments for improving maternal, neonatal, and child health and nutrition outcomes [14, 15]. In accordance with this, the Ethiopian government, in partnership with foreign partners such as UNICEF, has launched national media campaigns to promote preventive nutrition programs via radio, television, and social media [8, 16].

While these programs are substantial, their scope and impact have not been fully assessed. Assessing their performance is crucial for improving current methods, guaranteeing fair access, and guiding future program design. Despite significant expenditure in media efforts, actual evidence on their statewide efficacy in Ethiopia is limited. This study was thus designed to analyze the reach and impact of a statewide media campaign in Ethiopia, with the explicit goal of measuring changes in knowledge, attitudes, and practices related to critical nutritional services and preventative measures using a two‐round mobile survey.

## 2 Methods

### 2.1 Study Setting and Study Context Description

This study was carried out nationwide across all regions of Ethiopia between February and September 2023, in collaboration with VIAMO. UNICEF Ethiopia has been actively engaged in multiple areas of public health, with a particular emphasis on maternal and child health and nutrition. Within this framework, UNICEF launched a nationwide media campaign designed to raise awareness among mothers and caregivers about the importance of preventive maternal and child nutrition services. The campaign emphasized preventive nutrition interventions, particularly highlighting iron and folic acid (IFA) supplementation for pregnant women and routine growth monitoring for children. To ensure accessibility and inclusiveness, the campaign messages were disseminated in five widely spoken languages: Amharic, Afan Oromo, Tigrigna, Somali, and Afar, thereby reaching diverse population groups across the country. Recognizing the importance of evidence‐based programming, this study was undertaken to assess both the reach and the impact of the media campaign on preventive nutrition services in Ethiopia. Using a pre‐ and post‐evaluation design, the study aimed to generate insights into changes in knowledge, attitudes, and practices, providing critical evidence to inform future programmatic actions and policy decisions.

### 2.2 Study Design and Populations

The two‐round mobile survey employed a post‐comparison group evaluation design. It was conducted as a pre‐ and post‐assessment of the reach and impact of the nationwide media campaign on preventive nutrition services across all regions of Ethiopia. A total of 2,704 participants completed the survey, consisting of 2,058 males and 646 females from the general public, selected through quota sampling across different locations. Participants were enrolled and evaluated both at baseline and end‐line following the implementation of the media campaign. Eligible participants included mothers who were pregnant and/or had children, as well as their spouses or caregivers, provided they were able to give informed consent and respond to the survey via interactive voice response (IVR). Individuals unable to remain until the completion of data collection were excluded from the study.

Since the campaign was implemented nationwide, establishing a traditional control group unexposed to the intervention was not feasible. To address this, a post‐comparison group was created, consisting of participants who reported not being exposed to the campaign messages. This allowed for comparison of nutritional knowledge, attitudes, and practices between those exposed and unexposed. Because the media campaign was implemented nationwide, establishing a conventional control group that was unexposed to the intervention was not feasible. To address this, a post‐comparison group evaluation design was applied. At end‐line, participants were categorized based on their self‐reported exposure to the campaign messages: those who had been exposed formed the intervention group, while those who had not been exposed constituted the comparison group. Differences in knowledge, attitudes, and practices between the two groups were then assessed. To reduce bias and account for potential differences in background characteristics, propensity score matching was employed to strengthen the validity of the comparisons. In total, 2,704 respondents were randomly interviewed at both baseline and end‐line. For participant enrollment, potentially eligible respondents were contacted by phone and invited to participate through an IVR system managed by the investigators. Following oral informed consent, eligible individuals were included, and baseline data were collected. End‐line follow‐up data were subsequently obtained using the same procedure.

### 2.3 Intervention Description

The intervention was designed in alignment with the UNICEF nutrition intervention checklist, which addresses key nutrition‐related problems. The core messages of the campaign focused on promoting preventive nutrition services, specifically: iron and folic acid (IFA) supplementation during pregnancy, vitamin A supplementation for children, regular growth monitoring, deworming, and the importance of dietary diversity practices. The campaign was delivered through multiple communication channels to maximize coverage and reinforce message retention. These channels included radio spots, television broadcasts, and social media platforms, particularly the UNICEF Ethiopia Facebook page. Messages were disseminated repeatedly across the campaign period to ensure frequent exposure, although the exact number of exposures per participant varied depending on media access and engagement. The media campaign ran between February 2023 and September 2023, covering all regions of Ethiopia. To ensure cultural appropriateness and clarity, messages were pretested with target audiences before full implementation. They were then disseminated in five major local languages (Amharic, Afan Oromo, Tigrigna, Somali, and Afar) to reach diverse communities nationwide. Throughout the intervention, confidentiality of participants was maintained. Data were collected following a Random Digit Dialing (RDD) approach via mobile phones, and the investigators together with trained data collectors assessed both the reach and the impact of the campaign. Since establishing a traditional unexposed control group was not feasible due to the nationwide rollout, a post‐comparison group of participants who reported no exposure to the campaign was created to allow for impact evaluation.

### 2.4 Measurement and Variables

Assessing the reach and impact of the nationwide media campaign was the primary outcome of this study. The exposure variables included socio‐demographic factors as well as knowledge, attitude, and practice related to preventive nutrition services. Socio‐demographic characteristics considered were the person contacted, educational status, sex, call reach, and region.

Knowledge was assessed using questions with “yes” or “no” response options. Respondents were instructed not to guess but to select “I don't know” if they were unsure of the correct answer. Respondents were categorized as knowledgeable if they answered 50% or more of all knowledge questions correctly, and as not knowledgeable if they answered fewer than 50% correctly. Attitudes were measured similarly: respondents with 50% or more positive responses to attitude questions were categorized as having a positive attitude, while those with fewer than 50% were considered to have a negative attitude. We applied a ≥ 50% threshold to dichotomize knowledge, attitude, and practice scores because it represents the neutral midpoint of the scoring scale (i.e., respondents scoring at least half of the items correctly or positively were considered to have a basic or adequate level). This midpoint cut‐off was chosen to facilitate interpretation and communication of program reach to policymakers and program managers. To ensure robustness, sensitivity analyses were also conducted using alternative thresholds. Nutritional practice was defined as the respondent's use of preventive nutrition services at least once within the recommended period, measured with a “yes” or “no” format. For this study, reach referred to the extent to which the media campaign successfully connected with the intended audience across regions. Exposure to message referred to the frequency and intensity with which individuals came into contact with campaign messages. Impact was defined as the extent to which the campaign influenced knowledge, attitudes, and practices (KAP) related to preventive nutrition services.

### 2.5 Data Collection, Quality Management, Processing, and Analysis

The survey was conducted using mobile phone calls via Interactive Voice Response (IVR) technology. The calls were sent randomly every day to a list of phone number prefixes until the target was achieved. The steps to launch the surveys are: (1) question design and development; (2) script optimization; (3) translation and recording to five languages; (4) setup and testing; and (5) monitoring and data analysis. In addition, data were collected using designed and adapted, structured interviewer‐administered questionnaires during IVR. Questionnaires were translated into the local languages and then back translated to English by another person to maintain consistency. Trained data collectors conducted the process and supervised to monitor data quality. Closer supervision was undertaken during data collection. Before the real data collection, a pretest was done with 5% of the participants. Chronbach's alpha coefficient was used to estimate the reliability of the questionnaire, which was greater than 0.70. The results indicate robust internal consistency across all measures, ranging from 0.76 to 0.91, which well exceeds the standard threshold of 0.70. The questionnaires addressed diverse topics on nutrition and the interviews lasted 30 to 60 min for data collection. The collected data were analyzed by Stata version 17.

Prior to analysis, the data were checked for assumption, then analyzed and interpreted by the research team and a biostatistician. For pre‐ and post‐intervention comparison, a rate of media reach and impact at baseline and at the end‐line was compared using descriptive statistics and presented with tables and figures including a summary of the media campaign's reach. To identify independent predictors of media reach and impact on preventive nutritional practices a propensity score matching (PSM) analysis was conducted. Specifically, covariates included in the model were age, sex, education level, region of residence, and baseline knowledge, attitude, and practice scores. We applied the nearest matching method with a caliper width of 0.2 of the standard deviation of the logit of the propensity score, without replacement, to optimize balance between exposed and unexposed groups. Balance diagnostics (standardized mean differences) were checked to confirm covariate balance after matching.

### 2.6 Ethical Clearance and Consent to Participate

This study was conducted in full compliance with the ethical principles outlined in the Declaration of Helsinki for Medical Research involving human participants. Prior to the commencement of the study, formal approval was obtained from the Ethiopian Federal Ministry of Health (FMOH) research ethics committee with reference number MT/49/44/1243 and date 01/11/2023, ensuring that the research design and procedures adhered to the national ethical standards for human subject's research. Furthermore, additional permissions were secured from the respective health bureaus of all selected regions where the study was implemented, thereby ensuring institutional and regional endorsement of the research process. All prospective participants were provided with a clear and comprehensive explanation of the study objectives, purpose, and expected outcomes, ensuring that they fully understood the intent of the research before deciding whether to participate. Participants were also assured that their involvement was entirely voluntary and that they retained the right to withdraw at any time without facing any negative consequences. Since the study involved only non‐invasive data collection procedures such as structured interviews or questionnaires oral informed consent was deemed sufficient and was formally obtained from each participant before data collection. This approach was consistent with local ethical practices and standards for minimal‐risk research. To further safeguard the rights and well‐being of participants, confidentiality and anonymity were strictly maintained throughout the study. No personal identifiers were recorded, and all data were securely stored and used exclusively for research purposes. Data collection commenced only after each participant explicitly confirmed their willingness to participate, thereby reinforcing the principle of autonomy and voluntariness.

## 3 Results

### 3.1 Socio‐Demographic Characteristics of Participants

At the outset, a total of 718,877 phone numbers were generated using random digit dialing technology, and calls were initiated. Of these, 128,779 individuals answered, with 116,786 found to be eligible participants. Ultimately, a total of 2,704 individuals were approached and successfully completed the survey, resulting in an overall response rate of 100%.

Among the respondents, 79.1% (2,058/2,704) were male. Regarding educational attainment, 54.0% (1,461/2,704) had completed university education. The largest proportion of participants, 41.3% (1,117/2,704), were from the Oromia region (Table 1).

**Table 1:** Socio‐demographic characteristics of the participants who completed survey, *n* = 2704.

Variables	Category	Frequency (*n*)	Percentage (%)
IVA/RDD	Call sent	718,877	100.0
	Pick Up	124,779	17.4
	Eligible	116,786	16.3
	Started	31,081	4.3
	Completed	2704	0.4
Contacted Person	Care giver	1172	43.3
	Man with pregnant wife with no kids	262	9.7
	Man with pregnant wife with kids	997	36.8
	Pregnant with no kids	39	1.4
	Pregnant with kids	234	8.6
Reach	Yes	1157	42.8
	No	1547	57.2
Sex	Male	2058	79.1
	Female	646	23.9
Educational Status	No formal education	88	3.25
	Primary school	343	12.7
	Secondary school	812	30.0
	University	1461	54.1
Region	Oromia	1117	41.3
	Addis Ababa	702	25.9
	Amhara	376	13.9
	SNNPR	233	8.6
	Sidama	79	2.9
	Tigray	55	2.1
	Somali	41	1.5
	Afar	34	1.3
	Dire Dawa	29	1.1
	Benishangul Gumuz	23	0.8
	Gambela	11	0.4
	Harari	4	0.2

Abbreviations: IVR, Interactive Voice Response; RDD, Random Digit Dialing.

### 3.2 Nutritional Knowledge of the Participants

Table 2 presents the nutritional knowledge of participants across the country. At baseline, 86% of respondents had knowledge, which increased slightly to 88% at endline following the intervention. With regard to iron and folic acid (IFA) supplementation, 85% (2,297/2,704) of participants had knowledge at baseline, rising modestly to 87% (2,324/2,704) at endline. Similarly, knowledge of vitamin A supplementation improved from 91% (2,433/2,704) at baseline to 94% (2,539/2,704) at endline. For deworming medication, 72% (1,947/2,704) of participants had knowledge at baseline, increasing to 76% (1,433/2,704) at endline, reflecting a moderate gain in awareness of its benefits for preventing worm infestation. Knowledge of growth monitoring and promotion services remained consistently high, with 95% at baseline and 96% at endline. A detailed item‐specific analysis is provided in Table 2 (Table 2).

**Table 2:** Nutritional knowledge of the participants who completed survey, *n* = 2704.

Variables	Category	Pre‐ and post‐evaluation categories	
		Baseline number (%)	End line number (%)
Knowledge of IFA supplementation	Support fetal growth and maintain mothers’ health	351 (13%)	1162 (43%)
	Only support fetal growth	946 (35%)	0 (0%)
	Only maintain mother's health	946 (35%)	1135 (42%
	IFA is a determinant for health	54 (2%)	27 (1%)
	IFA has no benefit	108 (4%)	108 (4%)
	Don't know about IFA	297 (11%)	243 (9%)
	Knowledgeable	2297 (85%)	2324 (87%)
	Not knowledgeable	407 (15%)	351 (13%)
Knowledge of growth monitoring and promotion service	Checking for stunting	459 (17%)	459 (17%)
	Getting knowledge about parenting	1216 (45%)	1135 (42%)
	Access to vaccines and vitamins	892 (33%)	973 (36%)
	No benefit of growth monitoring	27 (1%)	27 (1%)
	I don't know about growth monitoring	108 (4%)	81 (3%)
	Knowledgeable	2567 (95%)	2567 (96%)
	Not knowledgeable	135 (5%)	108 (4%)
Knowledge of vitamin A supplementation	Avoid/prevent measles	595 (22%)	568 (21%)
	For improving vision	757 (28%)	838 (31%)
	To strengthen child immunity	1081(40%)	1135 (42%)
	I don't know	243 (9%)	163 (6%)
	Knowledgeable	2433 (91%)	2539 (94%)
	Not knowledgeable	243 (9%)	163 (6%)
Knowledge of deworming	Prevention of worm infestation	1487 (55%)	1433 (53%)
	Prevents anemia	460 (17%)	622 (23%)
	Don't know	541 (20%)	541 (20%)
	Other benefits	216 (8%)	108 (4%)
	Knowledgeable	1947 (72%)	2055 (76%)
	Not knowledgeable	757 (28)	649 (24)
Overall knowledge	Knowledgeable	2325 (86%)	2386 (88%)
	Not Knowledgeable	379 (14%)	318 (12%)

### 3.3 Nutritional Attitude of the Participants

The impact of the media campaign on attitude towards nutritional services of each type of nutrition was assessed and described in percentages. Table 3 shows attitude of the participants in various types of preventive nutritional services across the country. Accordingly, the study revealed that out of the study participants, 57% (1541/2704) had positive attitude towards nutritional intervention at the endline, showing there was a significant increase from the baseline, 52% (1406/2704)). The unfavorable attitude of the participants was decreased from 48% to 43% baseline to endline highlighting the importance of the intervention. In the baseline, around 59% (1595/2704) of participants agreed with the importance of regular growth monitoring. However, at the endline, it increased to 66% (1784/2704). The majority of participants in both surveys (60% vs. 62%) agreed with the importance of vitamin A supplementation. In both surveys, the highest proportion of respondents agreed on the importance of consuming deworming pills, followed by those who were neutral. At baseline, the majority of participants agreed that children should regularly receive deworming pills, whereas at endline the proportion of respondents expressing agreement was lower **(**Table 3 **).**

**Table 3:** Nutritional attitude of the participants who completed survey, *n* = 2704.

Variables	Category	Phases	
		Base line number (%)	End line number (%)
Overall attitude of IFA	Agree	668 (34%)	1663 (43%)
	Disagree	594 (24%)	514 (19%)
	Don't know about IFA	88 (8%)	162 (6%)
	Neither agree nor disagree	488 (34%)	365 (32%)
Overall attitude growth monitoring	Agree	1595 (59%)	624 (66%)
	Disagree	433 (16%)	118 (13%)
	Don't know about GMP	81 (3%)	21 (4%)
	Neither agree nor disagree	595 (22%)	153 (17%)
Overall attitude of vitamin A supplementation	Agree	1622 (60%)	570 (63%)
	Disagree	324 (12%)	103 (11%)
	Don't know about IFA	162 (6%)	49 (5%)
	Neither agree nor disagree	595 (22%)	193 (21%)
Overall attitude of deworming	Agree	1544 (56%)	1460 (54%)
	Disagree	401 (15%)	514 (19%)
	Don't know about IFA	189 (7%)	162 (6%)
	Neither agree nor disagree	622 (23%)	541 (20%)
Overall Attitude on preventive nutrition services	Positive	1406 (52%)	1541 (57%)
	Negative	1298 (48%)	1163 (43%)

### 3.4 Nutritional Practice of the Participants

The impact of the media campaign on participants’ nutritional practices was assessed and presented as percentages. Table 4 summarizes the practices related to different nutritional services across the country. Overall, there was an increase from baseline to endline in the proportion of respondents who reported consuming iron and folic acid (IFA) tablets and other micronutrient supplements. For example, growth monitoring practices remained unchanged (86% at baseline vs. 86% at endline), suggesting a possible ceiling effect **(**Table 4 **).**

**Table 4:** Nutritional practice of the participants who completed survey, *n* = 2704.

Variables	Category	Phases	
		Base Line number (%)	End line number (%)
Overall use of IFA supplementation among pregnant mothers.	Yes	1568 (58%)	584 (62%)
	No	1136 (42%)	954 (38%)
Overall practice of growth monitoring	Yes	2218 (86%)	2325 (86%)
	No	486 (18%)	379 (14%)
Overall practice of vitamin A supplementation	Yes	2055 (76%)	2163 (80%)
	No	649 (24%)	541 (20%)
Overall practice of deworming	Yes	1812 (67%)	1891 (70%)
	No	892 (33%)	813 (30)
Over all reach of the media campaign	Yes	1081 (40%)	1663 (43%)
	No	1622 (60%)	1041 (57%)

### 3.5 Predictors of Media Reach and Media Impact of Nutritional Services and Measures

Propensity score matching analysis was performed to assess the association of independent variables with the reach and impact of the media campaign on promoting preventive nutrition services and practices. The final analysis revealed that knowledge of deworming (proportion difference, 0.30; 95% CI: 0.20–0.49; *p* < 0.001), overall attitude toward growth monitoring (proportion difference, 0.70; 95% CI: 0.42–0.99; *p* < 0.001), overall attitude toward vitamin A (proportion difference, 0.50; 95% CI: 0.20–0.91; *p* < 0.001), and overall practice of IFA supplementation (proportion difference, 0.04; 95% CI: 0.02–0.09; *p* < 0.0001) were significant independent predictors of the reach and impact of the media campaign in promoting preventive nutrition services **(**Table 5 **).**

**Table 5:** Propensity score match analysis to know proportional difference between pre‐and post‐exposure of media campaign across the country, 2023.

Variables	Category	Exposed to media campaign (*n* = 2704)		Propensity score match analysis
		Baseline (%)	End‐line (%)	Standardized proportion difference (95% CI)
Knowledge of deworming	Prevention of worm infestation	1487 (55%)	1433 (53%)	0.30 (0.20, 0.49)
	Prevents anemia	460 (17%)	622 (23%)	
	Don't know	541 (20%)	541 (20%)	
	Other benefits	216 (8%)	108 (4%)	
Overall attitude growth monitoring (GMP)	Agree	1595 (59%)	624 (66%)	0.70 (0.42, 0.99)
	Disagree	433 (16%)	118 (13%)	
	Don't know	81 (3%)	21 (4%)	
	Neither agree nor disagree	595 (22%)	153 (17%)	
Overall attitude of vitamin A	Agree	1622 (60%)	570 (63%)	0.50 (0.20, 0.91)
	Disagree	324 (12%)	103 (11%)	
	Don't know	162 (6%)	49 (5%)	
	Neither agree nor disagree	595 (22%)	193 (21%)	
Overall practice of Iron and folic acid (IFA).	Yes	1568 (58%)	584 (62%)	0.04 (0.02, 0.09)
	No	1136 (42%)	954 (38%)	

Abbreviations: GMP, Growth Monitoring Program; IFA, Iron and folic acid.

## 4 Discussion

The influence of the media campaign message on nutritional knowledge, attitudes, and practices was assessed using interactive voice response (IVR) and Random Digital Dialing (RDD). Overall, media‐based interventions to improve nutritional services were effective, particularly in enhancing participants' general knowledge and attitudes. This finding is consistent with studies from other settings, which demonstrated that media education increases the likelihood of adopting specific health behaviors when messages are appropriately targeted [17, 18, 19, 20]. A possible explanation is that information dissemination, whether direct or indirect, positively influences behavior by raising awareness, shaping perceptions, and reinforcing healthy practices.

In the current study, 88% of participants were knowledgeable at endline compared to 86% at baseline, showing a modest improvement. This aligns with evidence from other media‐driven interventions, which found that targeting specific nutritional behaviors (such as breastfeeding promotion) significantly improved outcomes [19, 21]. A potential reason is that interventions designed to address a particular behavior are more likely to result in meaningful changes than broad, general media campaigns.

Attitude is widely recognized as one of the strongest and most immediate predictors of behavior [1]. In this study, 57% of participants demonstrated a positive attitude toward nutritional interventions at endline, compared to 52% at baseline. This improvement suggests that repeated reminders and exposure to campaign messages motivated participants to value healthy behaviors [1, 22, 23]. Prior research supports this finding, emphasizing that personalized or emotionally resonant communication enhances persuasiveness [22, 23, 24]. Furthermore, the proportion of participants with unfavorable attitudes decreased from 48% at baseline to 43% at endline, highlighting the positive influence of the intervention.

With regard to practice, there was a notable increase in the uptake of iron and folic acid (IFA) tablets and other micronutrient supplements from baseline to endline. Similar findings have been documented in previous studies, which showed that media campaigns promote engagement in specific health behaviors [25, 26]. However, only slight changes were observed for other nutrition‐related practices. This may be explained by structural and contextual barriers, such as low literacy levels, limited access to services, or insufficient intensity of messaging, which can constrain the impact of media‐based interventions.

Our study further revealed that exposure to media campaigns was consistently associated with slight but positive increases in adherence to dietary recommendations and use of nutritional services. Evidence from an Iranian study, which applied targeted communication during pregnancy, similarly demonstrated improvements in maternal well‐being [27]. Systematic reviews of media‐based interventions also corroborate that carefully designed campaigns can improve health‐related knowledge, attitudes, and behaviors [17, 19, 25, 28]. Specifically, our study found that knowledge of deworming was significantly associated with media exposure, in line with prior publications demonstrating that communication interventions increase awareness and uptake of preventive health measures [28, 29, 30].

Systematic reviews of nutrition interventions highlight that effectiveness depends not only on knowledge but also on the accessibility and availability of services [28, 31]. In our study, growth monitoring and vitamin A supplementation were significantly associated with media reach, while other practices were not. One possible explanation is that the intensity and dosage of interventions may have been insufficient to produce changes across all outcomes. Optimal intervention design often requires repetition, reinforcement, and targeted tailoring to sustain behavioral shifts, which a general media campaign may not fully achieve. Finally, while this study presents aggregate findings for the sample as a whole, it is important to acknowledge that the practical impact may vary across specific demographic subgroups. Factors such as sex, educational attainment, and regional or geographic contexts often influence nutrition. Although explicit subgroup analyses fell outside the scope of the current paper, future research should explore these potentially intersecting variables to tailor more localized and demographically targeted approaches.

As a strengths of the study, first, it was conducted nationwide, allowing findings to be generalized at the country level. Second, the use of electronic data collection enabled broad coverage across diverse regions, thereby strengthening the representativeness of the data and providing a clearer understanding of the impact of the media campaign. However, there are several limitations. First, a potential limitation of this study is the baseline sex imbalance within our participants, with a higher representation of male participants (79.1%). While this statistical adjustment minimizes internal bias, the skewed distribution may limit the generalizability of our findings to populations with a different sex composition, and future studies with a more balanced cohort are warranted to confirm these results across all demographics. Second, a limitation of this study is that exposure to the media campaign was evaluated based entirely on participant self‐report, which did not undergo external objective validation. Relying on self‐reported data introduces the potential for recall bias, as participants may over‐ or under‐report their exposure based on memory lapses or social desirability. Third, the observed changes after the campaign may not be solely attributable to the intervention and could have been influenced by confounding factors. Although participants were proportionally allocated by region, the number of respondents varied due to non‐response in certain areas, which may have introduced regional imbalances. Finally, although no other national nutrition campaigns were identified during the study period, we cannot entirely rule out the influence of other external communications that may have affected participants’ knowledge, attitudes, or practices.

## 5 Conclusion

In conclusion, this study showed that a nationwide media campaign using interactive voice response and random digital dialing led to modest but meaningful improvements in nutritional knowledge, attitudes, and practices. Gains were observed in areas such as iron and folic acid supplementation, vitamin A use, and deworming, while limited change in practices like growth monitoring may reflect ceiling effects or structural barriers. These findings align with global evidence that well‐targeted media interventions can promote healthier behaviors. However, while self‐report is a standard and practical approach for assessing reach in large‐scale media evaluations, these findings was interpreted with caution. Future research would benefit from incorporating digital analytics, verified ad‐impression metrics, or verified log data to complement self‐reported exposure and improve measurement accuracy. On top that, optimizing media dissemination by maximizing reach, escalating exposure intensity, and systematically reinforcing key messaging may yield greater intervention efficacy. Furthermore, the empirical insights gleaned from this evaluation offer a valuable framework to guide the design and implementation of future nutritional campaigns within Ethiopia and comparable low‐ and middle‐income countries.

## Author Contributions

**Feleke Doyore Agide:** methodology, software, data curation, investigation, validation, formal analysis, supervision, funding acquisition, visualization, writing – review and editing, writing – original draft, resources, project administration, conceptualization; **Beimnet Desalegn Kedida:** conceptualization, methodology, software, data curation, investigation, validation, formal analysis, writing – review and editing, writing – original draft, resources, project administration, visualization, funding acquisition, supervision; **Zeamanuel Anteneh Yigzaw:** conceptualization, methodology, software, data curation, investigation, validation, formal analysis, supervision, funding acquisition, visualization, project administration, resources, writing – original draft, writing – review and editing; **Yewubdar Hirpa:** conceptualization, methodology, software, data curation, investigation, validation, formal analysis, supervision, funding acquisition, visualization, project administration, resources, writing – original draft, writing – review and editing; **Eneyew Talie Fenta:** conceptualization, methodology, software, data curation, investigation, validation, formal analysis, supervision, funding acquisition, visualization, project administration, resources, writing – original draft, writing – review and editing; **Hiwot Getachew:** conceptualization, methodology, software, data curation, investigation, validation, formal analysis, supervision, funding acquisition, visualization, writing – review and editing, writing – original draft, resources, project administration; **Rachana Sharma:** conceptualization, methodology, software, data curation, investigation, validation, formal analysis, supervision, funding acquisition, visualization, project administration, resources, writing – original draft, writing – review and editing; **Nardos Birru:** conceptualization, methodology, software, data curation, investigation, validation, formal analysis, supervision, funding acquisition, visualization, project administration, resources, writing – review and editing, writing – original draft; **Stanley Chitekwe:** conceptualization, methodology, software, data curation, investigation, validation, formal analysis, supervision, funding acquisition, visualization, project administration, resources, writing – original draft, writing – review and editing; **Mussie Tefera:** conceptualization, methodology, software, data curation, investigation, validation, formal analysis, supervision, funding acquisition, visualization, project administration, resources, writing – original draft, writing – review and editing; **Finina Abebe Ware:** conceptualization, software, data curation, methodology, investigation, validation, formal analysis, supervision, funding acquisition, visualization, project administration, resources, writing – original draft, writing – review and editing; **Dessie Abebaw:** conceptualization, methodology, software, data curation, investigation, validation, formal analysis, supervision, funding acquisition, visualization, project administration, resources, writing – original draft, writing – review and editing; **Tolasa Yadate:** conceptualization, methodology, software, data curation, investigation, validation, formal analysis, supervision, visualization, funding acquisition, project administration, resources, writing – original draft, writing – review and editing.

## Funding

The authors have nothing to report.

## Conflicts of Interest

The authors declare no conflicts of interests.

## Transparency Statement

The lead author, Feleke Doyore Agide, affirms that this manuscript is an honest, accurate, and transparent account of the study being reported, that no important aspects of the study have been omitted, and that any discrepancies from the study as planned (and, if relevant, registered) have been explained.

## Data Availability

All relevant data are available from the corresponding author upon reasonable request. Corresponding author had full access to all of the data in this study and takes complete responsibility for the integrity of the data and the accuracy of the data analysis.
